# Effects of prolactin on the proliferation and hormone secretion of ovine granulosa cells *in vitro*

**DOI:** 10.5713/ab.23.0448

**Published:** 2024-04-26

**Authors:** Haiying He, Xiaohui Su, Huiguo Yang, Yingjie Zhang, Chunhui Duan, Ruochen Yang, Fengmei Xie, Yueqin Liu, Wujun Liu

**Affiliations:** 1 Department of Animal Science and Biotechnology, Xinjiang Agricultural University, Urumqi, Xinjiang 830052, China; 2 Moyu Bibang Sheep Industry Development Co. LTD, Hotan Prefecture, Xinjiang 848100, China; 3 Department of Animal Science and Biotechnology, Hebei Agricultural University, Baoding, Hebei 071000, China; 4 Animal Husbandry Institute, Xinjiang Academy of Animal Science, Urumqi, Xinjiang 830052, China

**Keywords:** Apoptosis, Granulosa Cells (GCs), Prolactin (PRL), PRL Receptor (PRLR), Proliferation

## Abstract

**Objective:**

The objective of this study was to investigate the effects of prolactin (PRL) on the proliferation and apoptosis of ovine ovarian granulosa cells (GCs) and the secretion of estrogen (E_2_) and progesterone (P_4_), as well as to explore the effects of PRL on related genes and proteins.

**Methods:**

We isolated ovarian GCs from 1-year-old small-tail Han sheep and identified PRL receptor (PRLR) on ovaries and follicle stimulating hormone receptor (FSHR) on ovarian GCs, respectively, using immunohistochemistry. PRL (0, 0.05, 0.50, 5.00 μg/mL) were added to GCs *in vitro* along with FSH, cell proliferation was measured by cell counting Kit-8 (CCK-8) and apoptosis by flow cytometry. The measurement of E_2_ and P_4_ content by enzyme-linked immunosorbent assays after 48 h and 72 h. The expression of functional genes and proteins was identified by real-time quantitative polymerase chain reaction (RT-qPCR) and Western-blot after 48 h.

**Results:**

PRLR was expressed in both follicular GCs and corpus luteum, whereas FSHR was expressed specifically. The proliferative activity was lower on day 1 while higher on day 4 and day 5. The apoptosis rate of GCs in the 0.05 μg/mL group was significantly higher than that in the control group after treatment with PRL for 24 h (p<0.05). Compared with the control group, the secretion of E_2_ in GCs was reduced significantly (p<0.05) in PRL treatment for 48 h and 72 h, while the secretion of P_4_ was significantly increased (p<0.05). The mRNA expression levels of *PRLR*, *FSHR*, *LHR*, *CYP11A1*, *HSD3B7*, and *STAR* were significantly higher than those in the control group (p<0.01), and the relative abundance of *BCL2* in all PRL group were increased after PRL treatment.

**Conclusion:**

PRL promoted the proliferation of GCs and supraphysiological concentrations inhibited apoptosis caused by down-regulation of *BAX* and up-regulation of *BCL2*. PRL inhibited E_2_ by down-regulating *CYP19A1* and promoted P_4_ by up-regulating *CYP11A1*, *STAR*, and *HSD3B7*.

## INTRODUCTION

Follicular development is the key to female reproduction, a complex multicellular process regulated by the endocrine system and the local microenvironment. As an important component and functional unit of the follicle, granulosa cells (GCs) play a key role in regulating follicular development from primordial follicle initiation to follicular maturation and ovulation. Numerous studies have shown that the proliferation and differentiation of GCs protect the growth of the oocytes, provide the living space for the oocytes, and the hormones secreted by GCs provide the power for the follicles to develop [1,2]. During each estrous cycle in female animals, only some of the follicles can mature and ovulate, while others stop developing or remain in a closed state. This phenomenon may be caused by the direct apoptosis of GCs caused by the transfer of hormonal information to the follicles. In most ruminants, only one or two follicles successfully ovulate in each oestrus cycle, which severely limits the development of animal husbandry. The mechanism of follicular development is complex and important. Therefore, it is important to do research on the crucial role of GCs function.

Prolactin (PRL) is a hormone associated with lactation [3], immunity [4], and growth [5] in females, and an increasing number of studies [6] have found that prolactin is directly related to follicular development and GCs function. PRL binds to prolactin receptors (PRLR) and was first identified by Rolland and Hammond [7] in 1975 on the surface of porcine GCs. The number of PRLR and the PRL specific binding capacity are reduced during follicular maturation. In the last few years, studies have found that PRL-L (prolactin-like protein, is a homolog of PRL in non-mammalian vertebrates and can act as a functional ligand of PRLR) transcript levels were highest in follicular walls of <2 mm follicles of chicken and progressively declined during follicle maturation [8]. There is also considerable evidence that PRL regulates the secretion of steroid hormones in GCs. PRL effectively up-regulated *PRLR* expression in GCs in the presence of follicle stimulating hormone (FSH). PRL suppressed FSH-induced estradiol (E_2_) production and increased FSH-induced progesterone (P_4_) production in GCs of rat [9]. E_2_ and P_4_ are the main ovarian steroid hormones, mainly synthesized by GCs, among which P450 cholesterol side chain lyase (encoded by *CYP11A1*), aromatase (encoded by *CYP19A1*) are key enzymes in their synthesis pathways. Three-beta-hydroxysteroid dehydrogenase (HSD3B7), reduced by PRL in chicken GCs, is a key enzyme in the pathway that produces P_4_ [10]. From these previous reports, we seem to know the general rule of prolactin on ovarian follicle development and GCs function in female animals, especially chickens, rats, etc., but for animals with seasonal estrus, the study of PRL on follicle development has not yet been characterized. Numerous studies [11] have shown that prolactin, along with melatonin, is greatly associated with seasonal estrus in female animals. The molecular mechanisms and potential roles of PRL in ovine follicular development remain largely unknown. Therefore, to investigate the formation mechanism of PRL on follicular development in seasonally estrus animals, we added different concentrations of PRL *in vitro* to observe the proliferation, apoptosis and hormone secretion of ovine GCs.

## MATERIALS AND METHODS

All procedures used in this study were approved by the Animal Care and Use Committee of Hebei Agricultural University (Approval number: 2022100) and Xinjiang Agricultural University (Approval number: 2022010).

### Immunohistochemical localization of prolactin receptor in ovine ovaries

The collected ovaries were cut lengthwise into two parts, placed in 4% paraformaldehyde (Guangnuo, Shanghai, China) and fixed for 24 hours. The fixed ovarian tissue was trimmed with a blade to approximately 1 cm^3^ and then dehydrated with graded concentrations of ethanol (Guangnuo, China), xylene (Guangnuo, China), made transparent and then immersed in wax (wax 12 h; wax II overnight). The wax-soaked tissue was embedded, placed in the freezer to cool, and sliced at a thickness of 4 μm. The cut wax pieces were placed in pure water at 45°C to spread the pieces. Finally, the pieces removed and baked in an oven at 42°C for 24 h. Afterwards, the slices were dewaxed with xylene and hydrated with graded concentrations of ethanol, the endogenous enzymes were eliminated with 3% H_2_O_2_ at room temperature for 10 min, the liquid was removed, and the slices were washed three times with phosphate-buffered saline (PBS) (Gibco, Grand Island, NY, USA). Slices were placed in sodium citrate at low fire for 20 min for thermal antigen repair, and after cooling to room temperature, the slides were removed and washed 3 times with PBS. Goat serum was added, put into a wet box and closed at room temperature for 1 h; the liquid discarded, a drop of Anti-PRLR mouse monoclonal antibody added (primary antibody; Abcam, Cambridge, MA, USA), overnight at 4°C; discarded the primary antibody, washed with PBS 3 times; added the Biotin × Polyclonal ovine anti-mouse immunoglobulin G (IgG) (secondary antibody; Bioss, Beijing, China) and put into a wet box at room temperature for 1 h, then discarded the secondary antibody, and washed with PBS 3 times; drops of DAB color developing agent (Zhuangmeng, Beijing, China) were added, and the color developing agent was kept at room temperature for 10 min. The color developing agent was then removed and washed 3 times with ddH_2_O. After restraining with haematoxylin, they were dehydrated with anhydrous ethanol for 3 min, cleared with xylene for 3 min, finally dried, sealed with neutral gum, photographed, and analyzed with Case Viewer (software, USA).

### Culture of ovine granulosa cells

All the reagents in this experiment were purchased from Gibco (USA), unless otherwise stated. Ovaries were obtained from thin-tailed Han sheep, 1 year, from Ruili slaughterhouse located in Tang County (Baoding, Hebei province). They were collected into normal saline supplemented with 1% penicillin and streptomycin and then transported to the laboratory within 3 h at 37°C. These ovaries were washed with 75% alcohol for 1 min, and then washed three times with Dulbecco’s phosphate-buffered saline (DPBS) to eliminate alcohol. Antral follicles (with good vascular supply and transparent follicular fluid) of ≥3 mm in diameter were dissected by sterile scalpels. The follicular fluid was pooled together, and GCs were collected immediately under centrifuging at 1,000×g for 10 min. All GCs were harvested and uniformly mixed into 50 mL sterile centrifuge tubes (Corning, Corning, NY, USA), these GCs were washed twice with culture medium and counted with a hemocytometer. Then, GCs were seeded in cell culture flasks at a density of 2×10^5^/well and cultured in DMEM/F12 supplemented with 10% fetal bovine serum (FBS), 1% streptomycin and penicillin mixture in a humidified atmosphere at 37°C and 5% CO_2_.

### Immunofluorescence assay

GCs were plated at a density of 3×10^5^ viable cells in Petri dishes (Nunc, Copenhagen, Denmark) with cell slides and cultured in a constant temperature (37°C) cell incubator for 24 h. When the cell confluence reached 70%, the old culture medium was aspirated, and the adherent cultured cells were slowly rinsed with PBS and fixed in Immunol Staining Fix Solution (Beyotime, Shanghai, China) for 10 min. The cells were permeated with 0.5% Triton for 10 min and blocked with 5% bovine serum albumin (Gibco, USA) for 1 hour at room temperature. Then the GCs were incubated with rabbit anti-follicle stimulating hormone receptor (FSHR) (No.2 Hormone Factory, Ningbo, China) antibody (22665-1-AP, China) at 4°C overnight. After primary antibody incubation, they were rinsed three times in PBS and then incubated with mouse anti-rabbit IgG/Bio antibody for 1 h at room temperature. After the GCs were washed in PBS to remove unbound secondary antibody, the cells were mounted on microscope slides with antifade mounting medium containing 4′, 6-diamidino-2-phenylindole (DAPI) (Solarbio, Beijing, China). Images were captured with a fluorescence microscope (BX-53; Olympus, Tokyo, Japan) and examined with a 40× objective.

### Prolactin treatment of granulosa cells

All the reagents in this experiment were purchased from Gibco (USA), unless otherwise stated. Primary GCs were cultured for 96 h in 10% FBS medium, digested with trypsin and collected into 50 mL sterile centrifuge tubes. Afterwards, the GCs were cultured with DMEM/F12 including 10% FBS in 6-well plates at a density of 1×10^6^/well and treated with different concentrations of PRL. Ovine PRL powder was purchased from ProSpec (CYT-240; ProSpec Telaviv Yafo, Israel). Normal saline was dissolved into a stock solution and the volume of stock solution was added according to the final concentration required so that the final concentrations of PRL were 0, 0.05, 0.50, 5.00 μg/mL. Numerous studies [12] have shown that the normal physiological concentration of PRL in sheep from estrus to the end of pregnancy is between 0.02 to 0.20 μg/mL, the lactation period may reach around 0.50 μg/mL. In this study, the concentration designed is used to determine the optimum concentration of PRL added to ovine GCs *in vitro*. And added 1 IU FSH and 0.1 μM androstenedione to GCs in each treatment group with 6 replicates.

### Cell proliferation assay

The viability of GCs was determined with the cell counting Kit-8 (CCK-8) (Ruixin, Quanzhou, China). Briefly, the cells were seeded into 96-well plates at a density of 1×10^4^ cells per well with 100 μL of culture medium including different concentrations of PRL (0, 0.05, 0.50, 5.00 μg/mL). GCs were treated continuously for 7 days, and 10 μL of CCK-8 solutions were added regularly every day for 1 h at 37°C. Then, the optical density (OD) values at 450 nm were measured by a microplate reader (Thermo, Waltham, MA, USA).

### Apoptosis assay

The extent of cell apoptosis was evaluated using flow cytometric analyses utilizing Annexin V-FITC/PI reagent (Beyotime, China) according to the manufacturer’s instructions. After PRL treatment of GCs (0, 0.05, 0.50, 5.00 μg/mL) for 24 h and 48 h, the cells were digested with trypsin substitute (without EDTA) for 3 min, and the digestion was stopped by adding DMEM/F12 (Gibco, USA) medium with FBS (Gibco, USA). The cells were gently blown down into a sterile 5 mL centrifuge tube (Corning, USA) and centrifuged at 1,000×g for 5 min. The cells were washed twice with PBS, and the supernatant was discarded after centrifugation (1,000 r/min for 5 min). Then 500 μL 1×binding buffer, 5 μL Annexin-V FITC and 5 μL PI were added gently to mix. The mixture was reacted at room temperature (25°C) in the dark for 15 min and detected by flow cytometry within 1 h.

### Hormone assays

Cell-culture supernatant concentrations of E_2_ and P_4_ were measured by respectively ovine estradiol (pg/mL) and progesterone (ng/mL) competitive enzyme-linked immunosorbent assays (ELISA) (Ruixin, China) according to the manufacturer’s instructions. After PRL treatment of GCs for 48 h and 72 h, supernatant was collected after centrifugation at 1,000 rpm for 10 min, within 30 min of collection and stored at −80°C until assay. The remaining cells were digested with Trizol for real-time polymerase chain reaction (PCR), which supernatant was collected after 48 h. Manufacturer supplied quality controls were used to ensure the reliability of the assay. Intra-assay and inter-assay coefficients of variation were both 10% for both assays. The E_2_ assay had a sensitivity of 1.0 pg/mL and the P_4_ of 0.1 ng/mL. Briefly, the assay procedure was as follows: standard wells with 50 μL standard substance in different concentrations and sample wells with 50 μL samples were set, and 100 μL of hrp-labeled antibody was added to each reaction well, 96-well plates were sealed with sealing plate membrane and incubated at 37°C for 1 h. Then plates were washed 5 times with washing solution and incubated at 37°C in dark for 15 min after adding 50 μL substrate A and B in each well. Added 50 μL terminating liquid to each well, and OD value of each well was determined at the wavelength of 450 nm with a microplate reader within 15 min. A standard substance linear regression curve with the standard product concentration as the abscissa and the corresponding OD value as the ordinate was drawn and the concentration value of each sample according to the curve equation was calculated.

### RNA extraction and real-time polymerase chain reaction

Total RNA was extracted from GCs treatment with different concentrations of PRL 48 h by using Trizol reagent. The concentration of RNA was quantified on a Nano Drop 2000 (Thermo, USA) at 260 nm absorbance. RNA (100 ng) was reversed transcribed using RT-PCR kit (Takara, Dalian, China) to remove genomic DNA (gDNA Eraser, up to 1 μg/reaction, 2 min, 42°C) and to reverse transcribe (Master Mix, 37°C for 15 min, 85°C for 5 s) the RNA samples following manufacturer’s instruction. Quantitative real-time PCR (qRT-PCR) was performed to detect relative abundance of mRNA. Primers were designed using Oligo software 6.0. Relative abundances of *PRLR*, *FSHR*, *LHR*, *CYP19A1*, *CYP11A1*, *HSD3B7*, *STAR*, *BAX*, *BCL2*, *TP53*, and *CASP3* mRNA were determined using the ABI 7300 Real-Time PCR system (Applied Biosystems, Carlsbad, CA, USA) with 480 SYBR Green I Master Kit (Roche Applied Science, Penzberg, Germany), using the following program: 95°C for 5 min; 40 cycles of 94°C for 30 s, 60°C for 30 s and 72°C for 30 s; and 72°C for 6 min). The ovine-specific primers for target genes are listed in Table 1. The relative abundances of mRNA for the target genes were normalized based on the mRNA transcript abundance of the house-keeping gene, *GAPDH*, relative quantification of target-gene mRNA abundances was determined using the 2^−ΔΔCT^ method.

### Western blot assay

Total proteins from tissues and cells were lysed conforming to the user’s guidebook of radio immunoprecipitation assay lysis buffer (Beyotime, China); this was followed by separation with 10% sodium dodecyl sulfate-polyacrylamide gel electrophoresis (SDS-PAGE) and transfer with polyvinylidene difluoride membranes (Bio-Rad, Hercules, CA, USA). After that, membranes were subjected to a standard blocking with 5% non-fat milk, hybridization with primary antibodies at 4°C overnight, and incubation with secondary antibodies at room temperature for one hour. The bands were detected according to the instructions of the ECL detection kit (Santa Cruz Biotechnology, Santa Cruz, CA, USA). The primary antibodies are PRLR, FSHR, LHR, CYP19A1, CYP11A1, HSD3B7, STAR, BCL2, BAX, TP53, and CASP3 (Abcam, Cambridge, UK), and goat anti-rabbit IgG (H+L) secondary antibody (Abcam, UK).

### Statistical analysis

Six replicates were performed for each experiment procedure. All Statistical analyses were performed using SPSS 22.0 (USA). Independent sample T-test was used to analyze the secretion content of E_2_ and P_4_ in GCs after PRL treatment for different times. One-way analysis of variance was used for other multiple data and the data are presented as mean± standard deviation (SE). Differences were considered statistically significant at the levels of p<0.05. All drawings were made using GraphPad Prism 9.0 (software, USA).

## RESULTS

### Results of immunohistochemical staining

**Figure 1:**
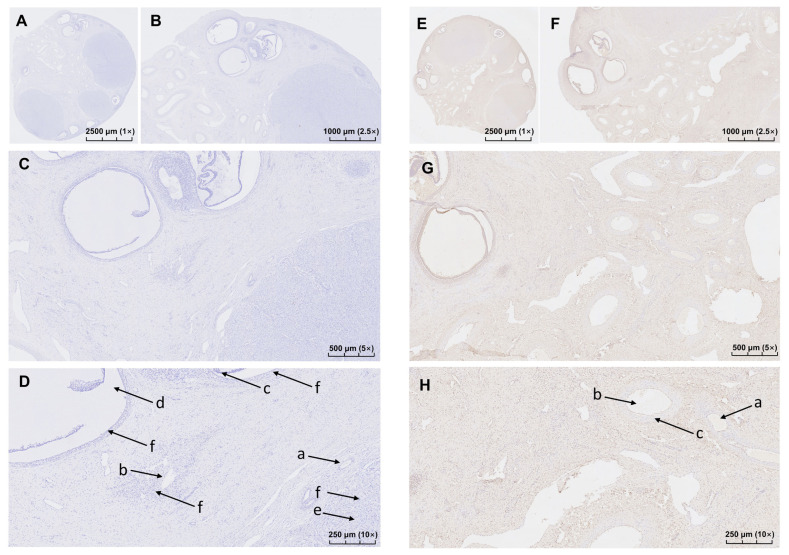
Immunohistochemical localization of PRLR in ovine ovaries. (A)–(D) were positive and (E)–(H) were negative controls. (A) and (E). 1×field of vision; (B) and (F). 2.5×field of vision; (C) and (G). 5× field of vision; (D) and (H). 10× field of vision; a. primary follicle; b. secondary follicle; c. theca cells; d. cavity of follicle; e. corpus luteum; f. granulosa cells. PRLR, prolactin receptor.

The positive immunoreaction products were brown with clear contrast, and the positive cells were easy to identify. PRLR was expressed in follicular GCs and corpus luteum at all levels, but not in the ovarian stroma (Figure 1). And PRLR expression was high in GCs layer.

### Identification of ovine granulosa cells

**Figure 2:**
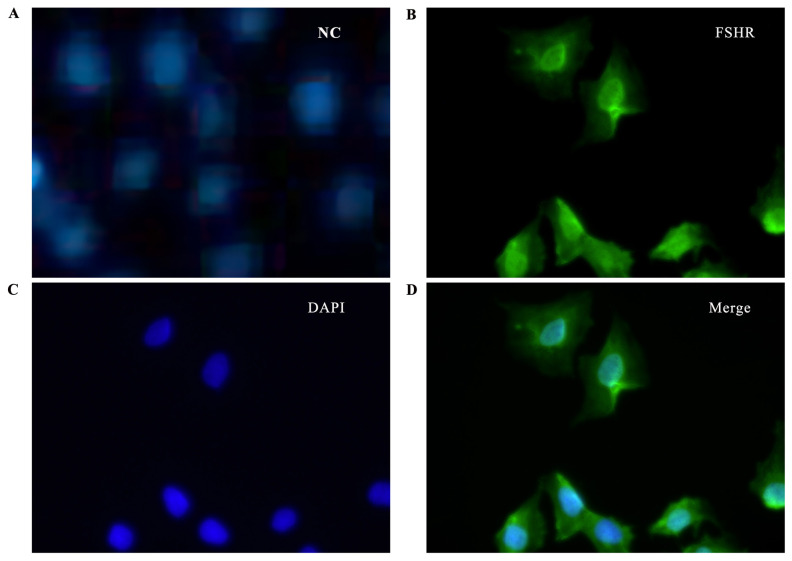
Identification of sheep GCs. The green marker indicates cells expressing FSHR, and the blue marker indicates 4′, 6-diamidino-2-phenylindole DAPI-stained nuclei. Merge is a green fluorescently labeled FSHR with a blue fluorescently labeled DAPI overlay. GCs, granulosa cells; FSHR, follicle stimulating hormone receptor; DAPI, 4′, 6-diamidino-2-phenylindole; NC, negative control.

To elucidate the potential role of PRL in GCs differentiation, the distribution and expression of ovine GCs were identified. The expression of FSHR, a specific marker of GCs in the ovary, was detected with immunofluorescence (Figure 2). The green fluorescent-labeled cells are FSHR-positive cells and blue regions are DAPI-stained nuclei. Merge provides an overlay of the green fluorescently labeled FSHR and the blue fluorescently labeled DAPI, denoting high purity of the GCs.

### Effects of prolactin treatment on the proliferation of granulosa cells

**Figure 3:**
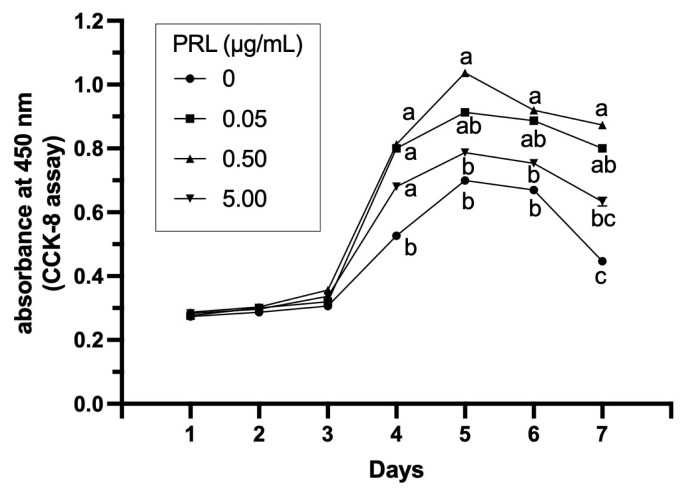
The growth curve of ovine GCs in 7 days. OD value of each well was determined at the wavelength of 450 nm, and the greater the number of cells, the greater the OD value. ^a–c^ In the same day, the same letter indicate difference was not significant (p>0.05), values with different letters indicate significant difference (p<0.05). GCs, granulosa cells; OD, optical density.

The cell growth curve is shown in Figure 3 of the treatment with PRL for different concentrations (0, 0.05, 0.50, 5.00 μg/mL) on GCs for 7 days. The proliferative activity of GCs in each treatment group had the same trend; the activity was lower on day 1 while higher on day 4 and day 5, and then decreased after day 6. The GCs activity of all the experimental groups (0.05, 0.50, 5.00 μg/mL) were significantly higher than the control group at day 4 (p<0.05). From day 5 to day 7, the GCs activity of 0.50 μg/mL PRL group was significantly higher than that of control group (p<0.05). The other two groups (0.05, 5.00 μg/mL) were basically higher than the control group, but the difference was not significant (p>0.05).

### Effects of prolactin treatment on the apoptosis of granulosa cells

**Figure 4:**
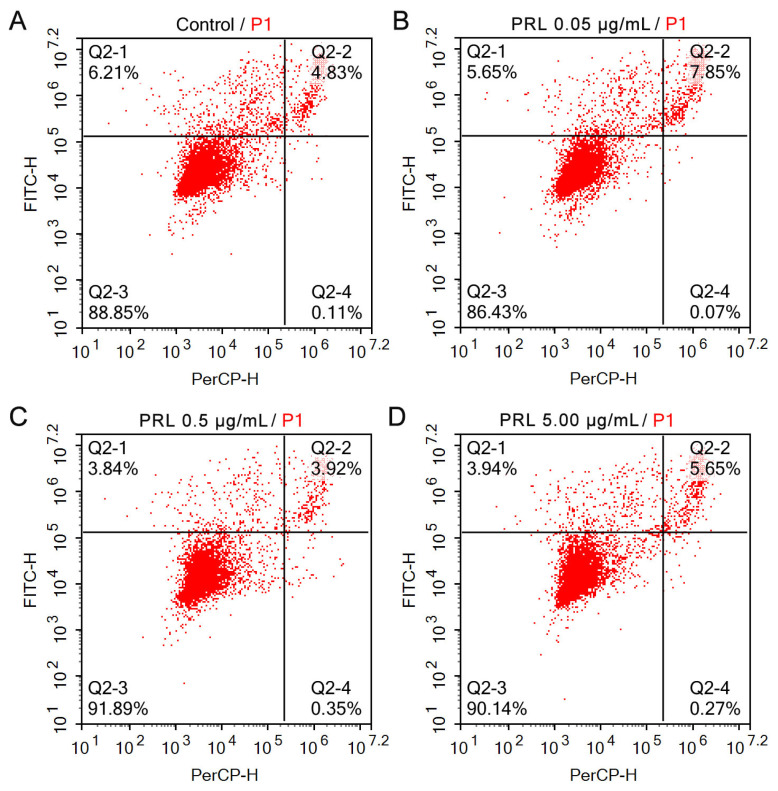
Treatment with PRL on apoptosis in cultured ovine GCs. Q2-1: early apoptotic cells; Q2-2: late-stage apoptotic cells; Q2-3: normal living cell; Q2-4: dead cells. PRL, prolactin; GCs, granulosa cells.

Treatment with PRL of different concentrations (0, 0.05, 0.50, 5.00 μg/mL) on GCs for 24 h and 48 h, and then staining Annexin V-FITC/PI is shown in Figure 4, the apoptotic cells (early and late apoptosis), normal living cells and dead cells were distinguished. The results of GCs apoptosis rate by Annexin V-FITC/PI detection are shown in Table 2.

The apoptosis rate of GCs in the 0.05 μg/mL group was significantly higher than that in the control group after treatment with PRL for 24 h (p<0.05), the apoptosis rate of 0.50 μg/mL group and 5.00 μg/mL group were significantly reduced (p<0.05), the apoptosis rate of 0.50 μg/mL group was significantly lower than that in 5.00 μg/mL group (p<0.05). The apoptosis rate of GCs in 0.05 μg/mL group, 0.50 μg/mL group, and 5.00 μg/mL group was significantly reduced compared with the control group after 48 h treatment with PRL (p<0.05), in which, the apoptosis rate of 0.05 μg/mL group and 5.00 μg/mL was significantly lower than that in 0.50 μg/mL group (p<0.05), the apoptosis rate of 5.00 μg/mL group was significantly lower than that in 0.05 μg/mL group (p<0.05).

### Effects of prolactin on E_2_ and P_4_ secretion in granulosa cells

Compared with the control group, the secretion of E_2_ in GCs was reduced significantly (p<0.05) in PRL treatment for 48 h and 72 h, while the secretion of P_4_ in GCs was significantly increased (p<0.05), as shown in Table 3. Meanwhile, the secretion of E_2_ at 72 h was higher than that at 48 h (p<0.05), both in the control group and treatment group. On the contrary, the secretion of P_4_ at 48 h was higher than that at 72 h (p<0.05). We can see the effect of PRL on the hormone secretion of the GCs, namely the inhibition of E_2_ and the stimulation of P_4_. This effect is not altered by increasing the concentration of PRL but is influenced by the concentration. There is no significant difference between the effect of 0.50 μg/mL and 5.00 μg/mL group.

### Effects of prolactin on relative abundances of hormone genes and proteins (*PRLR*, *FSHR*, *LHR*, *CYP19A1*, *CYP11A1*, *HSD3B7*, *STAR*) in granulosa cells

**Figure 5:**
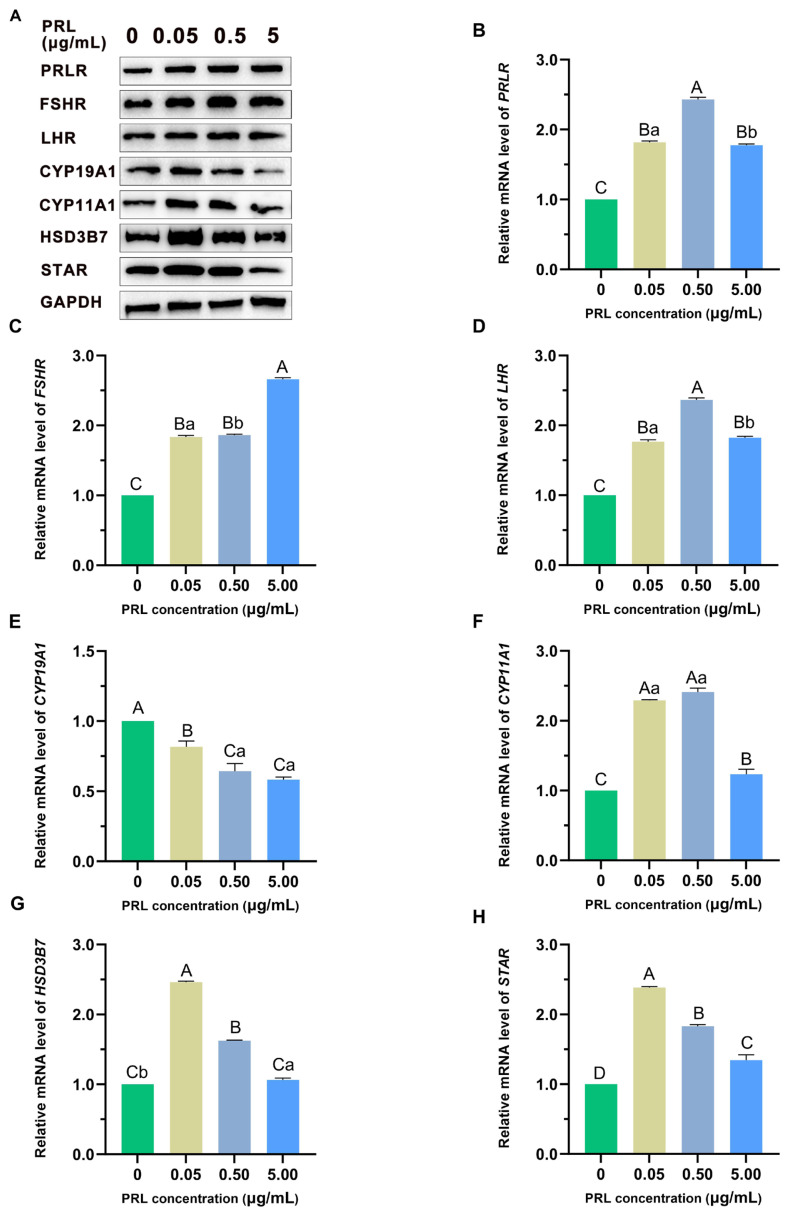
Effect of treatment with different concentrations of PRL on the mRNA expression of GCs function-related proteins (A) and genes (B–H), there are *PRLR*, *FSHR*, *LHR*, *CYP19A1*, *CYP11A1*, *HSD3B7*, and *STAR*. The different capital letters indicate that the difference is significantly (p<0.01); The different lowercase indicate that the difference is significant (p<0.05); the same letter mark indicates that the difference is not significant (p>0.05). PRL, prolactin; GCs, granulosa cells.

The relative abundance of *PRLR*, *FSHR*, *LHR*, *CYP19A1*, *CYP11A1*, and *HSD3B7*, *STAR* mRNA and proteins of ovine GCs treated with PRL for 48 h is shown in Figure 5. After PRL supplementation, the mRNA expression levels of *PRLR*, *FSHR*, *LHR*, *CYP11A1*, *HSD3B7*, and *STAR* in GCs were significantly higher than those in the control group (p<0.01). Including, *PRLR* and *LHR* mRNA expression was significantly higher (p<0.01) in the 0.50 μg/mL group (Figure 5B and 5D), FSHR mRNA expression was significantly higher (p< 0.01) in the 5.00 μg/mL group (Figure 5C), *HSD3B7* and *STAR* mRNA expression was significantly higher (p<0.01) in the 0.05 μg/mL group (Figure 5G and 5H). The mRNA expression level of *CYP19A1* was significantly lower than that of the control group (p<0.01), 0.50 μg/mL group and 5.00 μg/mL group were significantly lower than 0.05 μg/mL group (p<0.01) (Figure 5E). It was concluded that different concentrations of PRL affect the expression of steroid hormone-related genes, which may be the cause of changes in E_2_ and P_4_ secretion in GCs.

### Effect of prolactin on the relative abundance of apoptotic genes and proteins (*BCL2*, *BAX*, *TP53*, and *CASP3*) in GCs

**Figure 6:**
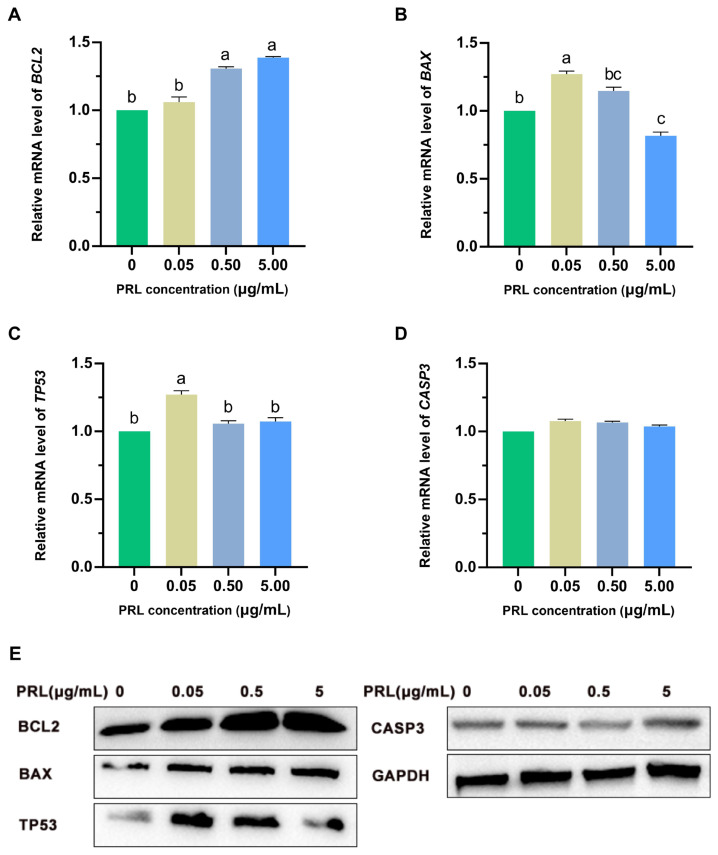
PRL treatment affected mRNA expression of apoptosis-related genes (A–D) and proteins (E) in ovine GCs, there are *BCL2*, *BAX*, *TP53*, and *CASP3*. The different capital letters indicate that the difference is significantly (p<0.01); The different lowercase indicate that the difference is significant (p<0.05); the same letter mark indicates that the difference is not significant (p>0.05). PRL, prolactin; GCs, granulosa cells.

The relative abundance of *BCL2*, *BAX*, *TP53*, and *CASP3* mRNA and proteins of ovine GCs treated with PRL at different concentrations (0, 0.05, 0.50, and 5.00 μg/mL) for 48 h are shown in Figure 6. Compared with the control group, the relative abundance of *BCL2* mRNA in all PRL group were increased after PRL treatment, in which 0.50 μg/mL group and 5.00 μg/mL group were significantly higher than those in control group and 0.05 μg/mL group (p<0.05), and there was no significant difference between 0.50 μg/mL and 5.00 μg/mL or between control group and 0.05 μg/mL (p>0.05) (Figure 6A). The relative *BAX* mRNA in 0.05 μg/mL group was of significantly higher abundance than that in control group, 0.50 μg/mL group and 5.00 μg/mL group (p<0.05), in which 5.00 μg/mL group was significantly lower than that in control group (p<0.05), and there was no significant difference between 0.50 μg/mL group and control group or 5.00 μg/mL group (p>0.05) (Figure 6B). The expression pattern of *TP53* was like that of *BAX*, the relative abundance of *TP53* mRNA in 0.05 μg/mL group was significantly higher than that in the control group, 0.50 μg/mL group, and 5.00 μg/mL group (p<0.05) (Figure 6C). There was no significant difference in the relative expression of *CASP3* mRNA between the control group and the experimental group (p>0.05) (Figure 6D).

## DISCUSSION

During normal follicular development, a mature antral follicle grows from a primordial follicle containing a single oocyte surrounded by GCs. Normal proliferation and steroidogenesis of GCs is essential for oocyte growth, maturation and fertilization, and subsequent embryonic development. PRL is mainly secreted by the pituitary gland, and a reproductive hormone related to animal estrus, which mainly regulates ovulation in female animals through the gonadal axis. Although previous studies found that the ovaries also secrete a small amount of PRL, which can directly affect follicle development [13], but PRL, especially large dose, induces the act of GCs steroid hormone synthesis which remains unknown. Our hypothesis that PRL doses affected the proliferation and steroidogenesis of GCs and that there are some regulatory relationships between PRL and steroidogenesis is supported by the study results. The results indicated that the concentration of PRL affected the proliferation of ovarian GCs and the secretion of steroid hormones, and as the concentration increased, the effect of large and supraphysiological doses of PRL became more pronounced. Therefore, this highlighted the importance of exploring interactions between PRL and the secretion of steroid hormones, which may provide new perspectives on the molecular mechanism of high dose PRL affecting ewe reproduction and the pathology of the development of hyperprolactinemia.

In 2008, Gómez-Brunet et al [14] reported on the endogenous periodicity of ovarian activity and changes in PRL secretion in ewes under long solar cycles. He found that the concentration of PRL in sheep was around 0.40 μg/mL in June and then changed periodically with light intensity over a year, reaching its lowest level in December, around 0.02 μg/mL, this indicated that the change of PRL concentration controls seasonal reproductive activity in sheep. On this basis, to further investigate the effect of PRL on ovine follicular development, our study further complemented the function of GCs at supraphysiological concentrations (5.00 μg/mL) of PRL. We found that as PRL levels increased, GCs proliferation and apoptosis first increased and then decreased, indicating that high PRL levels inhibit follicular development. In addition, E_2_ and P_4_ decreased and increased with increasing PRL concentration, suggesting that PRL inhibits E_2_ secretion and stimulates P_4_ secretion, and the supraphysiological dose of PRL seriously affected the function of GCs. Studies have confirmed that PRL regulates oestrus in sheep through the FSH signaling pathway under the influence of melatonin and other endocrines [15]. Therefore, further work is required to verify the molecular mechanism of PRL-induced GCs differentiation and steroid hormone secretion.

The growth and development of follicles are closely related to the proliferation and differentiation of ovarian GCs. And GCs play an important role at each stage of follicle development from the initial to the mature. GCs will proliferate and differentiate accompanied with FSHR after follicle initiation, at this time, follicular development and GCs proliferation are regulated by hypothalamus-pituitary-gonadal axis hormone, and studies have shown that GCs proliferation and maintain cell cycle can be promoted by FSH activating pi3K and Smad2/3 pathways [16]. Therefore, in this study, an equal amount of FSH was added to each treatment group to better observe the effect of PRL on GCs proliferation. In recent years, studies have reported that in addition to FSH and luteinizing hormone (LH), PRL is also secreted by the pituitary gland and can also act as an important hormone of growth factor that plays a role in cell proliferation and differentiation. For example, PRL can promote the proliferation of dairy cow mammary epithelial cells, B lymphocytes and trophoblastic cells in early pregnancy. PRL has also been reported to promote GCs proliferation in conjunction with other hormones such as auxin [17], however, there are few reports on the direct effect of PRL on GCs proliferation.

PRL secreted by the pituitary gland in animals binds specifically to PRLR on target tissues to initiate signal transduction, and the presence of PRLR on ovaries was confirmed by immunohistochemistry in our experiment. And FSHR is specifically present on GCs. In this study, we found the proliferation of GCs was promoted by adding PRL alone in cell culture medium, and the expressions of *FSHR* and *PRLR* mRNA were upregulated. This suggests that PRL can act as a growth factor or messenger to stimulate the growth of GCs because of PRL increasing the stimulation of GCs to FSH signals. However, it does not mean that cell proliferation will always increase with higher PRL dose; there is an optimal concentration of GCs for PRL stimulation. In our study, PRL of 0.50 μg/mL was the optimal growth concentration of ovine GCs. Some researchers believed that the effect of PRL on GCs proliferation might be related to the degree of cell differentiation, the mitotic rate of GCs increased with the increase of PRL concentration in 3 to 5 mm follicles of cows, while PRL had no obvious effect on the proliferation of GCs in 6 to 10 mm follicles [18]. Therefore, the effect of the proliferation of GCs on high concentration of PRL was lower than that of the medium concentration in this study; we speculated that the proliferation rate might be slowed down due to the promotion of cell differentiation by supraphysiological concentration of PRL.

A large part of follicles on the ovary of female animals become atretic during development. Although the specific process of atretic follicles at different stages is different, the ultrastructure of atretic follicles is found to be the first to appear in GCs, vacuoles and atretic bodies. Therefore, it is widely believed that oocytes lose their protective effect due to the apoptosis of ovarian GCs, which eventually leads to follicular atresia. Current studies have shown that there are many factors affecting GCs apoptosis, among which hormones are the most important micro-environmental factor for follicular atresia. Both *in vitro* and *in vivo* experiments have confirmed that both FSH inhibit the apoptosis of GCs, and the sharp decrease in the number of FSHR after ovulation leads to the apoptosis of GCs [19].

In addition to gonadotropins such as FSH and LH, which inhibit apoptosis of GCs, PRL, as an important pituitary hormone secreted in animals, also participates in apoptosis. Current studies have shown that PRL not only exerts an effect on apoptosis of neuronal cells, but also finds that PRL is closely related to follicular atresia [20]. At present, there are different opinions about whether PRL promotes or inhibits apoptosis of GCs. It has also been reported that PRL has anti-apoptotic effect on GCs, Perks et al [21] found that adding C2-ceramide to human ovarian GCs would lead to apoptosis but adding PRL to human ovarian GCs could significantly reduce the level of apoptosis, and the presence of PRL obviously inhibited GCs apoptosis. In this study, different from the above results, 0.05 μg/mL PRL promoted the apoptosis of GCs after being treated with PRL for 24 h, while 0.50 μg/mL and 5.00 μg/mL PRL significantly reduced the apoptosis of GCs. We mentioned earlier FSH can inhibit the GCs apoptosis, in this experiment, PRL caused the increase of *FSHR* expression, indicating that PRL may affect GCs apoptosis by mediating FSH signal. However, the apoptosis of GCs was inhibited after treatment with all concentrations of PRL for 48 h, indicating that the effect of PRL at low concentration on GCs apoptosis was short-term.

Ovarian GCs apoptosis-related genes roughly divided into two categories, one type is to promote cell apoptosis genes, such as *BAX*, *TP53*; another type is to inhibit cell apoptosis genes, such as the *BCL2*, the *BCL2* and *BAX* genes belong to the same family, *BAX/BCL2* has been generally accepted is the key factor of apoptosis or not. BAX protein can change the permeability of mitochondrial inner membrane of cells, release cytochrome C and other pro-apoptotic factors, and induce caspase activation. Meanwhile, TP53 protein increases, preventing cell cycle changes and exacerbating cell apoptosis. In this study, medium and high concentrations of PRL upregulated *BCL2* gene expression, downregulated *BAX* expression and thus inhibited granulocyte apoptosis. However, the effect of low concentrations of PRL on *BAX/BCL2* was exactly the opposite, suggesting that PRL may affect granulocyte apoptosis through the *BAX/BCL2* pathway. In addition, it was observed that the expression levels of *FSHR* and *BCL2* mRNA were basically the same as the concentration of PRL increased. Whether PRL induces changes in the intensity of *BAX/BCL2* signaling through FSH needs to be further investigated.

Follicular growth and development are influenced by autocrine or paracrine hormones and growth factors from GCs, follicular membrane cells and oocytes. GCs not only provide nutrients to oocytes and regulate their growth but are also the major production sites for E_2_ and P_4_ and other important reproductive hormones. Numerous studies have shown that PRL causes changes in the secretion of steroid hormones in ovary GCs of mammals and birds. Wang et al [22] added 1 μg/mL PRL into the GCs culture medium of rats to inhibit the secretion of E_2_ significantly. Nakamura et al [9] inhibited E_2_ production and stimulated FSH to induce P_4_ production after PRL was added into the co-culture system of rat oocytes and GCs. The research results of this study are basically consistent with those of the above scholars; the secretion of E_2_ decreases caused by PRL, while the secretion of P_4_ increases with the concentration and duration of PRL treatment. This indicated that the addition of PRL to ovine GCs cultured *in vitro* can inhibit the secretion of E_2_ and promote the secretion of P_4_. Some scholars believe that the effect of PRL on the secretion of P_4_ in GCs is related to the degree of cell differentiation, which needs further verification [23].

To analyze the mechanism of PRL affecting steroid hormone secretion in GCs, we detected the expression of many genes related to hormone secretion. Studies have shown that PRL has a high binding ability to PRLR in undifferentiated chicken follicular GCs, and can activate PI3K signaling pathway, and the binding ability gradually decreases with the maturity of follicles [24]. The secretion of E_2_ and P_4_ in GCs is not only affected by the key enzymes of the synthesis pathway, but also correlated with the upstream signal transduction regulation of the hypothalamus pituitary gonadal axis. FSHR exists on the membrane of GCs, and binds FSH. Its functions are to activate aromatase, which affects the secretion of E_2_ and P_4_ and the expression of related genes in GCs; the other is to induce the formation of LHR, and LH induces the production of androsterone or testosterone. Under the action of aromatase, androsterone or testosterone is converted into E_2_. Sites et al [25] had shown that adding FSH to GCs cultures increased the transcription strength of FSHR, 1 IU/mL of FSH was added to each treatment group in this study to amplify the effect of PRL, and the relative expression level of *FSHR* gene was significantly increased compared with the control group and increased with the increase of PRL dose. The results showed that adding PRL to the GCs culture medium could promote the binding of FSH and FSHR, thus affecting the secretion of steroid hormone in GCs.

Aromatase is a rate-limiting enzyme for E_2_ synthesis, and the main gene encoding aromatase is *CYP19A1*, the activation of FSHR usually leads to the production of cAMP and AKT signals, thus affecting the expression of aromatase genes. In the presence of FSH and cAMP activator, Krasnow et al [26] added PRL to rat ovarian GCs, inhibited E_2_ secretion and decreased *CYP19A1* expression, which was consistent with the results of this experiment. Steroid rapid regulatory protein (STAR) is an important regulatory factor in the process of anabolic steroid hormones, closely linked to the transmembrane transport of cholesterol. STAR can participate in the secretion of P_4_ and influence the secretion of T induced by luteinized GCs. The regularity of *STAR* expression can be used as an indicator of corpus luteum function. P450 cholesterol side chain lyase, encoded by the *CYP11A1* gene, and 3β-hydroxysteroid dehydrogenase, encoded by *HSD3B7*, are the main catalytic enzymes for the synthesis of P_4_ [27,28]. The former synthesizes cholesterol into 17-acetylitestosterone, and the latter further synthesizes P_4_. The results of this study showed that PRL could significantly improve the level of *STAR* mRNA, but with increasing PRL dose, the expression level of *STAR* first increased and then decreased, and the promoting effect of low mass concentration (0.05 μg/mL) of PRL was most obvious. This is not entirely consistent with the results of Anuradha and Krishna [29] who treated bats with embryonic developmental delay with PRL (0.50, 1.00 μg/mL), which alleviated the symptoms of P_4_ ovarian insufficiency and significantly increased the expressions of *PRLR*, *LHR*, *STAR*, and *HSD3B7*. In this experiment, the expression levels of *PRLR* and *STAR* first increased and then decreased after the gradient addition of PRL, which may be because the high concentration of PRL promoted the proliferation of GCs, led to an increase in ERK phosphorylation, decreased the expression levels of *PRLR* and *STAR*, and inhibited the secretion of E_2_. The addition of PRL significantly increased the gene expression levels of *CYP11A1* and *HSD3B7*, which was consistent with the changes in P_4_ secretion in GCs. These results suggest that PRL regulates P_4_ secretion by affecting the expression of *CYP11A1* and *HSD3B7* in GCs.

In a word, PRL up-regulated the expression of *PRLR*, *FSHR*, and *LHR* in FSH-containing ovine GCs medium expanded FSH signaling, led to the downregulation of *CYP19A1* to inhibit E_2_ secretion, and upregulated the expression of *CYP11A1* to promote P_4_ secretion. Our study provides a basic theory for the functional changes in GCs affected by PRL in female animals.

## Figures and Tables

**Table 1:** Ovine specific primer sequences for real-time polymerase chain reaction

Gene	Full name	Forward primer sequence (5′-3′)	Reverse primer sequence (5′-3′)	Fragment size (bp)
*GAPDH*	Glyceraldehyde-3-phosphate dehydrogenase	CTGACCTGCCGCCTGGAGAAA	GTAGAAGAGTGAGTGTCGCTGTT	149
*PRLR*	Prolactin receptor gene	CCCCTTGTTCTCTGCTAAACCC	CTATCCGTCACCCGAGACACC	163
*FSHR*	Follicle-stimulating hormone receptor gene	AATGATGTTTTCCAGGGAGC	TGCTGCTTGCTTTTTAGTCC	140
*LHR*	Luteinizing hormone receptor gene	ATCCAGAGCTGATGGCTACC	GCAGCTGAGATGGCAAAGAA	115
*CYP19A1*	Cytochrome P450 family 19 subfamily A member 1	TCGTCCTGGTCACCCTTCTG	CGGTCTCTGGTCTCGTCTGG	187
*CYP11A1*	Cytochrome P450 family 11 subfamily A member 1	GTTTCGCTTTGCCTTTGAGTC	ACAGTTCTGGAGGGAGGTTGA	120
*STAR*	Steroidogenic acute regulatory protein gene	GGTGCTGAGTAAAGTGATCC	CATCTCCTCGTAGAGTGTGA	127
*HSD3B7*	3 beta-hydroxysteroid dehydrogenase gene	GGAGACATTCTGGATGAGCAG	TCTATGGTGCTGGTGTGGA	485
*BAX*	BCL2 associated X, apoptosis regulator	TTCCGACGGCAACTTCAACT	CTGATCAACTCGGGCACCTT	102
*BCL2*	BCL2 apoptosis regulator gene	GGGGTCATGTGTGTGGAGAG	TACAGCTCCACAAAGGCGTC	143
*TP53*	Tumor protein p53	TTCCCCTTCCCTCAACAAGC	CCTCACAACCTCCGTCATGT	145
*CASP3*	Caspase 3 gene	TTCAGAGGGGACTGTTGCAG	CAGTCCAGTTCTGTGCCTCG	83

**Table 2:** Effect of different concentrations of PRL on apoptosis of ovine GCs^1)^

PRL (μg/mL)	0	0.05	0.5	5
24 h apoptosis rate (%)	11.04±0.01^b^	13.50±0.02^a*^	7.76±0.02^d^	9.59±0.01^c*^
48 h apoptosis rate (%)	10.56±0.03^a^	6.71±0.05^c^	7.28±0.09^b^	6.13±0.03^d^

SEM, standard error of the means; PRL, prolactin; GSs, granulosa cells.

1) Independent sample T-test was used to analyze the apoptosis of GCs after PRL treatment for different times. Comparisons between the different groups based on a multivariate analysis of variance (ANOVA).

a–d In the same row, the same letter indicate difference was not significant (p>0.05), values with different letters indicate significant difference (p<0.05). In the same column,

* indicate significant difference (p<0.05).

**Table 3:** Effect of PRL on E_2_ secretion (pg/mL) and P_4_ secretion (ng/mL) in ovine GCs^1)^

Time	Term							

	E_2_ (pg/mL)				P_4_ (ng/mL)			
	
	0	0.05	0.50	5.00	0	0.05	0.50	5.00
48 h	304.92±6.83^a^	283.45±3.22^a^	285.66±7.86^b^	263.82±4.17^b^	5.14±0.43^c^	6.82±0.47^b^	7.31±0.49^a^	7.46±0.34^a^
72 h	382.37±7.17^a*^	302.79±9.56^b*^	296.34±6.29^b*^	283.65±8.12^c*^	7.91±0.76^c*^	8.64±0.29^b*^	10.36±0.87^a*^	10.56±0.67^a*^

SEM, standard error of the means; PRL, prolactin; E_2_, estrogen; P_4_, progesterone; GSs, granulosa cells.

1) Independent sample T-test was used to analyze the secretion content of E_2_ and P_4_ in GCs after PRL treatment for different times. Comparisons between the different groups based on a multivariate analysis of variance (ANOVA).

a–c In the same row, the same letter indicate difference was not significant (p>0.05), values with different letters indicate significant difference (p<0.05). In the same column,

* indicate significant difference (p<0.05).
